# Expanding Horizons in Craniomaxillofacial Reconstruction: The Role of Exoscopic Microsurgery in Head and Neck Surgery

**DOI:** 10.3390/cmtr19010010

**Published:** 2026-02-03

**Authors:** Khalid Abdel-Galil, Kemal Mustafa Tekeli

**Affiliations:** Sheikh Tahnoon Bin Mohamed Medical City, Al-Ain, Abu Dhabi P.O. Box 15258, United Arab Emirates

**Keywords:** exoscope, microsurgery, head and neck reconstruction, free flap, robotics, surgical ergonomics

## Abstract

Exoscopic systems are increasingly used as an alternative to the operating microscope in microsurgical reconstruction, offering high-definition visualisation, shared operative viewing, and greater flexibility in surgeon positioning. This retrospective case series describes the use of exoscopic visualisation during microsurgical reconstruction in five illustrative head and neck and reconstructive cases. Different commercially available exoscopic platforms were utilised, and feasibility, workflow integration, and surgeon-perceived ergonomic aspects were assessed descriptively. Exoscopic visualisation was feasible for completion of microvascular anastomoses across a range of complex reconstructions. From the surgeons’ perspective, exoscopy allowed a more flexible working posture during prolonged microsurgical tasks and may offer advantages in training environments, particularly for junior surgeons. Further studies incorporating objective outcome measures are required to better define the role of exoscopy in microsurgical practice.

## 1. Introduction

Microsurgical head and neck reconstruction demands precision, depth perception, and prolonged operating times, necessitating superior visualisation tools. Traditionally, the operating microscope (OM) has been regarded as the gold standard for microvascular reconstruction. However, prolonged use of the operating microscope has been associated with significant ergonomic challenges for surgeons, particularly sustained neck flexion, shoulder elevation, and static postures during lengthy microsurgical procedures. Multiple studies have reported a high prevalence of work-related musculoskeletal disorders among microsurgeons, including chronic neck and lower back pain, which may negatively impact surgeon well-being and career longevity. These ergonomic limitations have driven growing interest in alternative visualisation platforms that allow a more neutral posture and dynamic positioning of the surgical team [1,2]. Exoscopy, particularly advanced 3D 4K systems, has gained increasing interest due to improved ergonomics and high-resolution imaging [3,4,5]. Initially developed for neurosurgical applications [4], exoscopic systems are now increasingly utilised in craniomaxillofacial (CMF) and reconstructive surgery [6,7,8,9,10].

## 2. Methodology

This work was conducted as a retrospective case series examining the use of exoscopic visualisation during microsurgical reconstruction in head and neck & free tissuereconstructive surgery. Cases in which an exoscope was used for the microvascular component of reconstruction were identified from operative records and reviewed. Five cases were included. These cases were selected to illustrate the use of exoscopic systems in a range of complex reconstructive scenarios requiring microsurgical free tissue transfer. The intention was not to create a comparative or consecutive cohort but to demonstrate feasibility and practical application across different anatomical regions and reconstructive demands. All procedures were performed by experienced microsurgeons. Exoscopic systems were employed during microvascular anastomoses. The choice of exoscopic platform varied between cases and reflected availability at the time of surgery and institutional resources. In one case, both an exoscope and a conventional operating microscope were used during reconstruction. All systems provided high-definition three-dimensional visualisation. Clinical data were collected retrospectively from patient medical records and operative notes. Information recorded included patient demographics, diagnosis, type of resection and reconstruction, and the exoscopic platform used. Ergonomic aspects, including surgeon posture, comfort during prolonged microsurgical tasks, and perceived musculoskeletal strain, were assessed descriptively based on the operating surgeons’ experience. No objective ergonomic assessment tools or validated scoring systems were used. Ethical approval for the study was obtained from the appropriate institutional review board. All procedures were performed in accordance with the Declaration of Helsinki. Written informed consent was obtained from all patients for inclusion in the study and for the use of anonymised clinical data and clinical images for publication.

## 3. Case Vignettes

### 3.1. Case 1

A 66-year-old male underwent subtotal mandibulectomy with bilateral neck dissections for a pT4aN0M0 moderately differentiated squamous cell carcinoma with free fibula osseoseptocutaneous flap reconstruction in February 2023.

**Figure 1:**
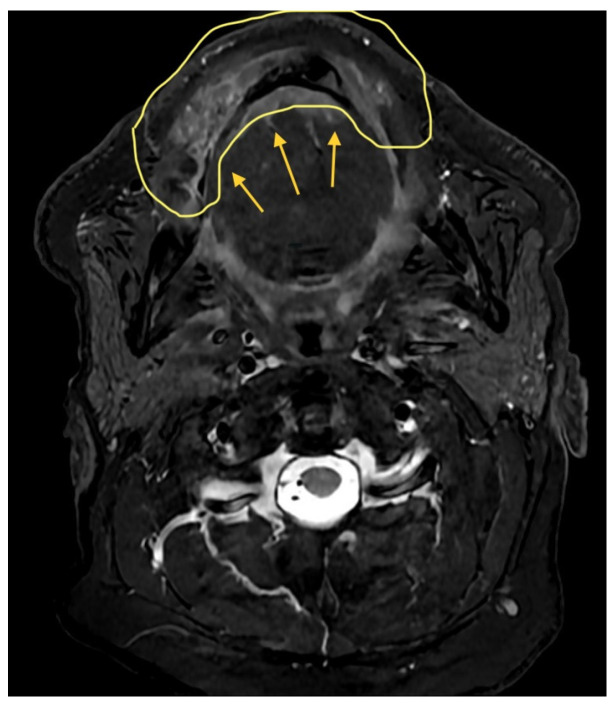
Case 1—Preoperative MRI. Yellow arrows and circle indicate the primary tumor extension.

**Figure 2:**
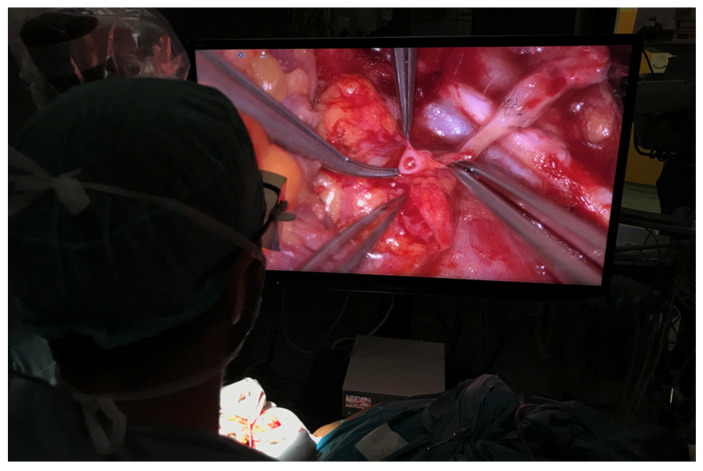
Case 1—Intraoperative exoscopic view.

**Figure 3:**
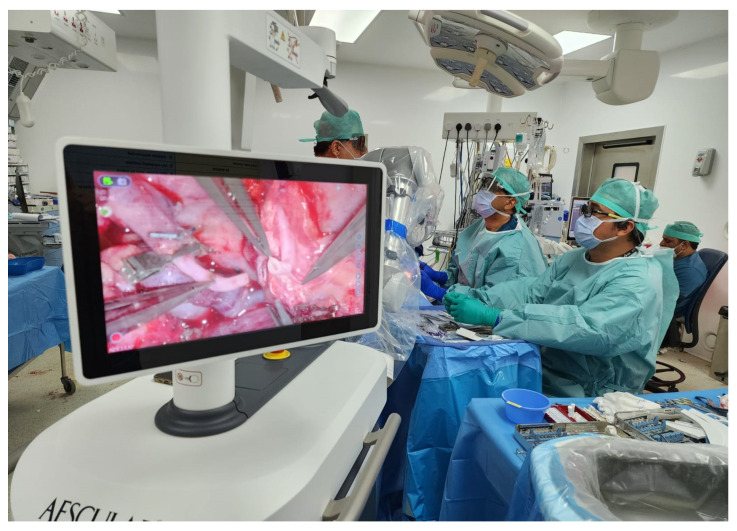
Case 1—Intraoperative exoscopic setup.

The Aesculap Aeos robotic digital exoscope (Aesculap Inc., Lehigh County, PA, USA) was used during microvascular anastomoses providing excellent depth perception and ergonomics as well as allowing simultaneous visualization for assistants, operating room staff and trainees (Figure 1, Figure 2 and Figure 3). The exoscopic setup also permitted a more neutral working posture during the microsurgical phase.

### 3.2. Case 2

**Figure 4:**
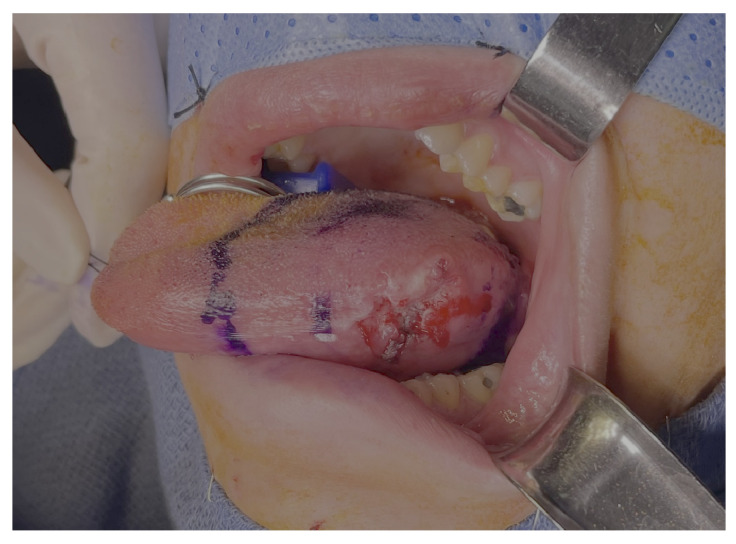
Case 2—Tongue Tumor surgical marking.

**Figure 5:**
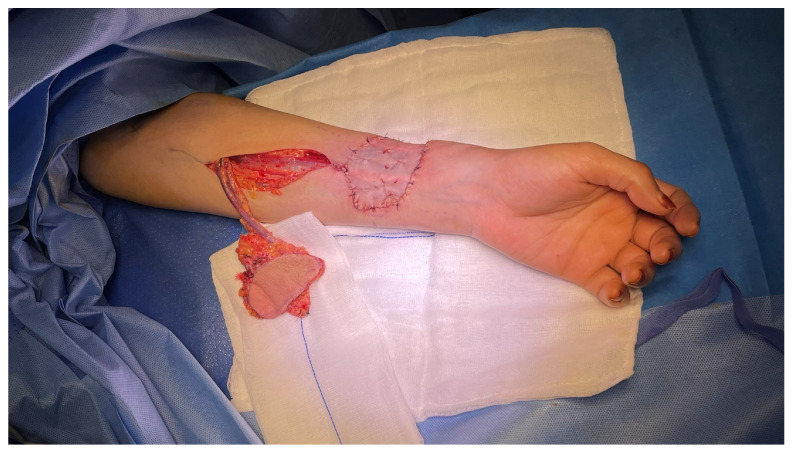
Case 2—Completed Forearm flap harvest.

**Figure 6:**
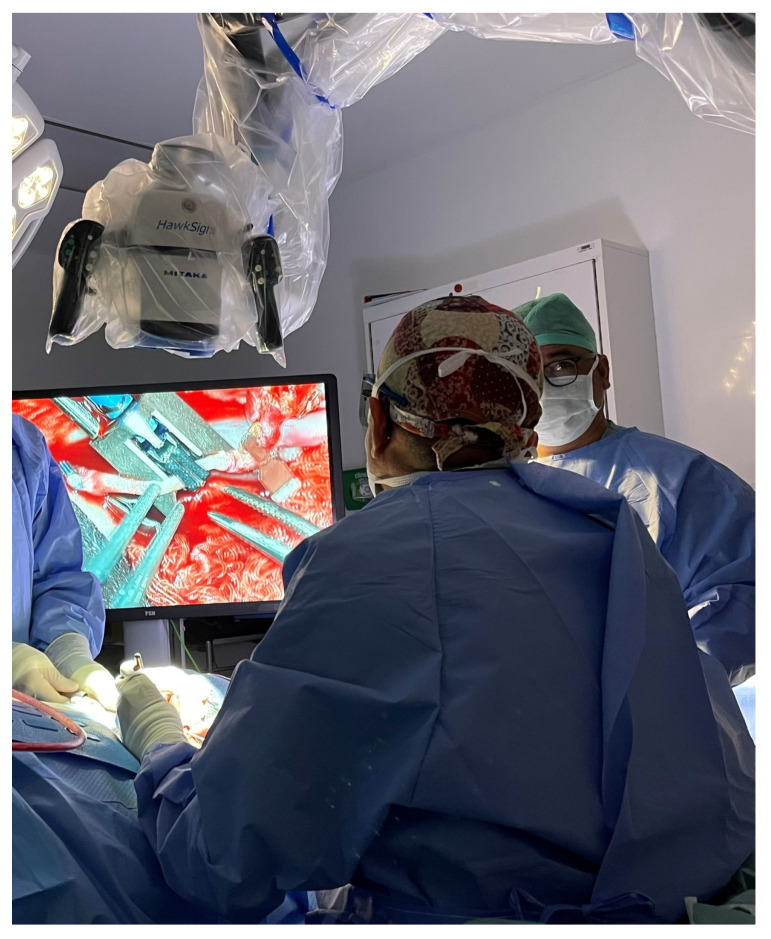
Case 2—Mitaka exoscope in use during microsurgery.

**Figure 7:**
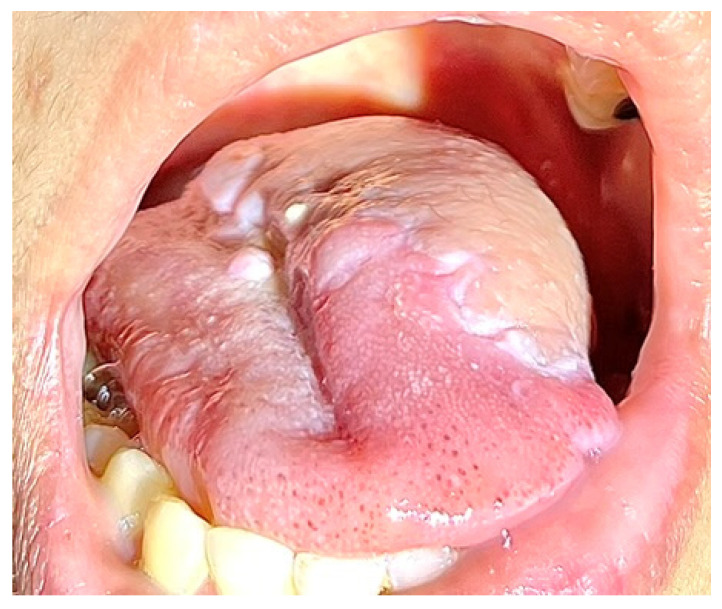
Case 2—Postoperative status at 3 months.

A 37-year-old female underwent extended hemiglossectomy with ipsilateral neck dissection in April 2024 for a pT3N1M0 (DOI 17mm) moderately differentiated squamous cell carcinoma. Immediate reconstruction was undertaken using a modified non-dominant radial forearm fasciocutaneous free flap using the Mitaka Hawksight platform (Mitaka USA Inc., Wheat Ridge, CO, USA) for microsurgery (Figure 4, Figure 5, Figure 6 and Figure 7).

Adequate illumination and visual clarity facilitated successful microvascular anastomoses without reverting to the OM.

### 3.3. Case 3

**Figure 8:**
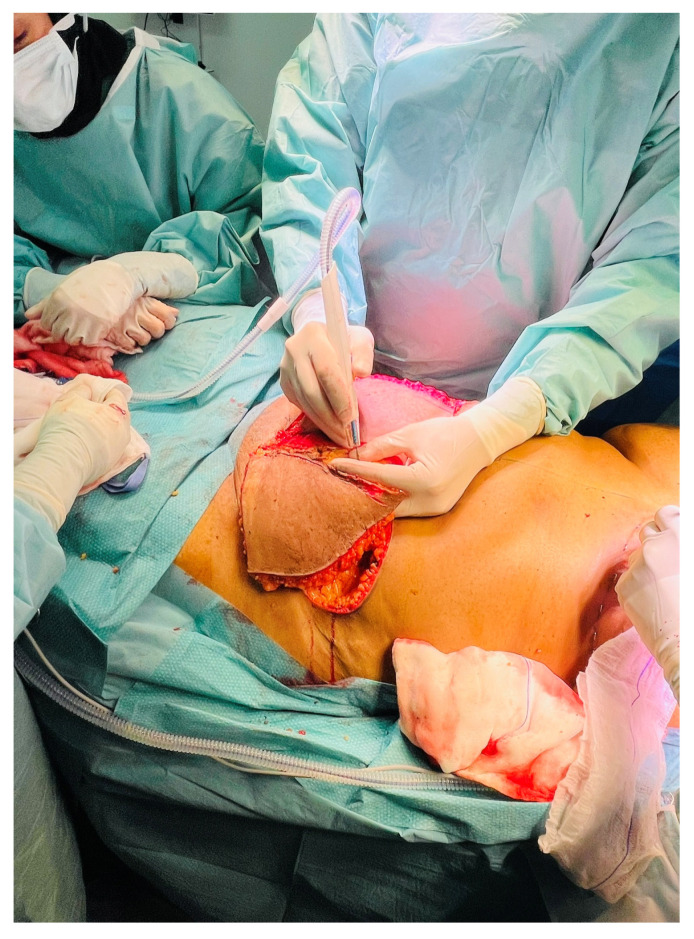
Case 3—DIEP flap harvest.

**Figure 9:**
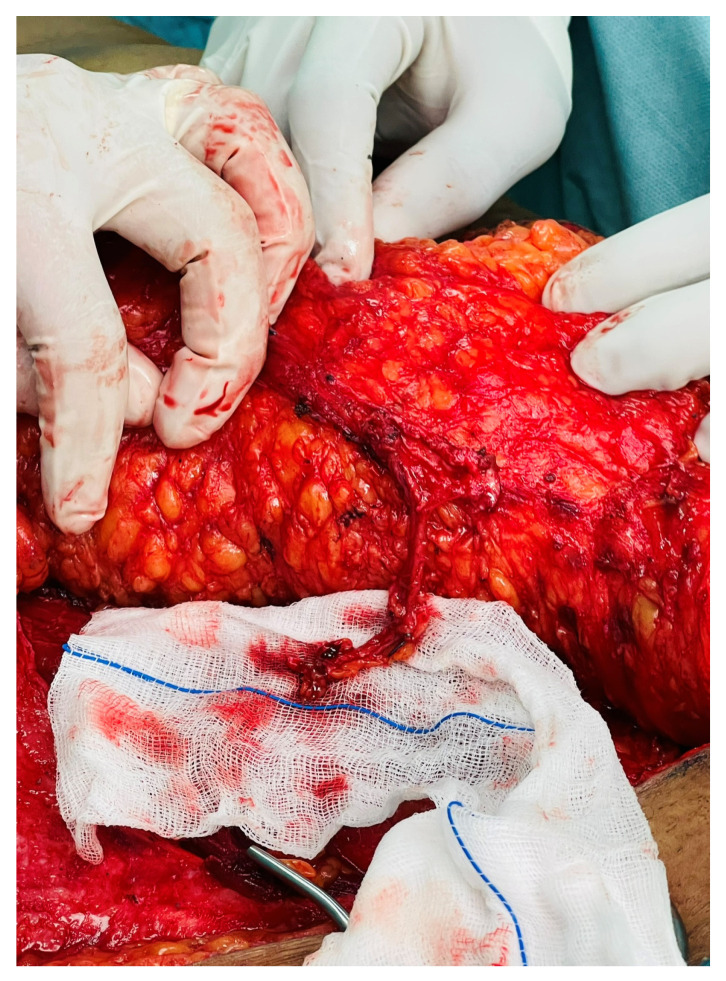
Case 3—completed flap harvest showing vascular pedicle.

**Figure 10:**
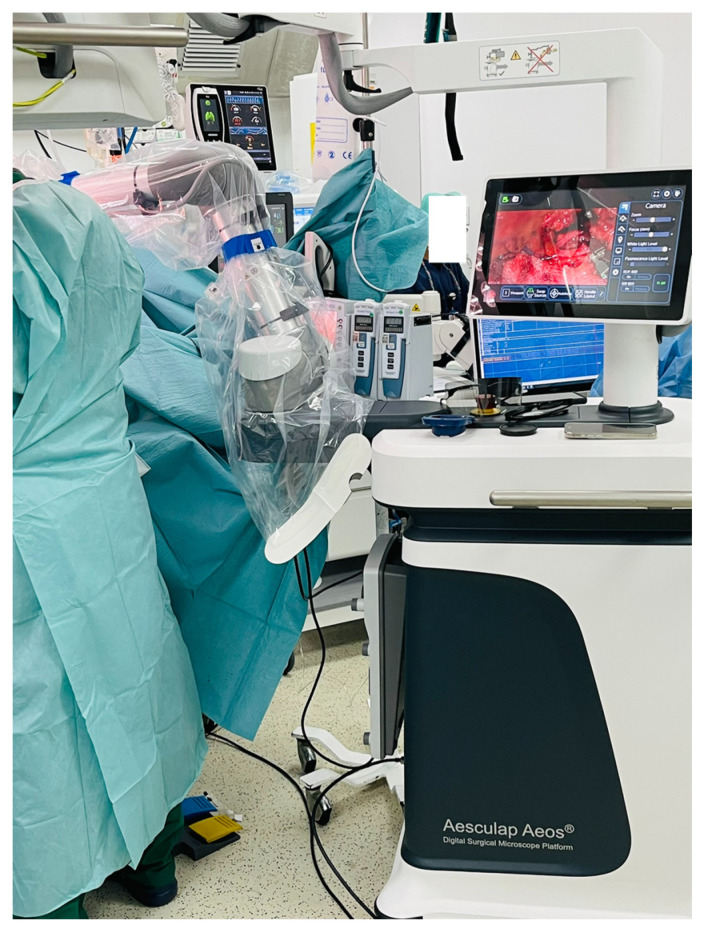
Case 3—Aeos exoscope in use intraoperatively.

A 41-year-old female underwent secondary post-mastectomy bilateral breast reconstruction using deep inferior epigastric perforator (DIEP) flaps in May 2024. Microsurgical anastomoses to the internal mammary vessels were performed using the Aesculap Aeos exoscope following appropriate chest wall access. Although operative time was slightly longer compared with conventional microscope-assisted procedures, image stability was satisfactory, and the operating surgeons noted a more comfortable working posture during the microsurgical phase (Figure 8, Figure 9 and Figure 10).

### 3.4. Case 4

**Figure 11:**
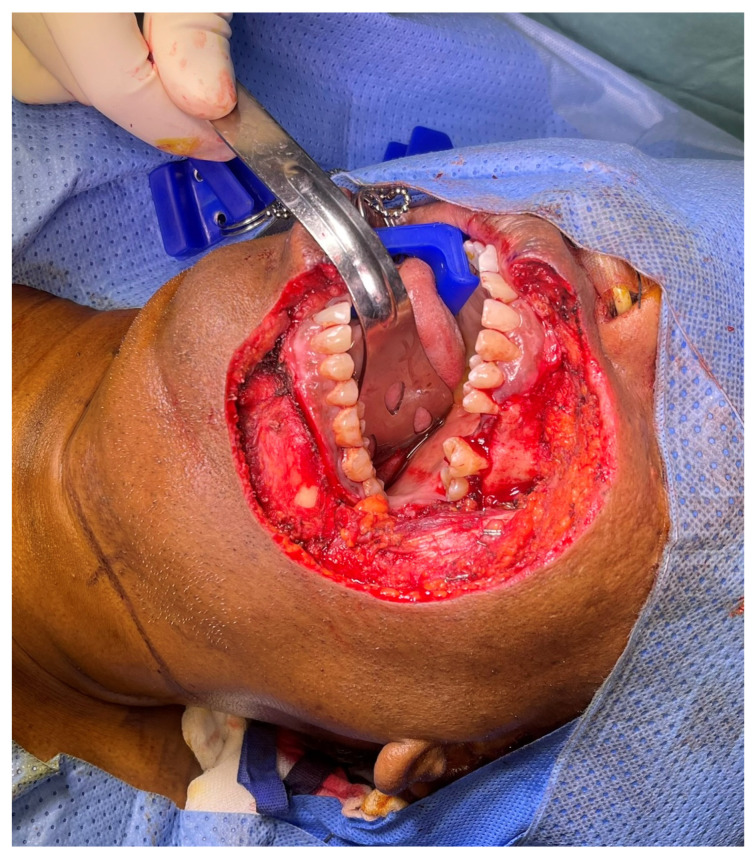
Case 4—Ablative defect.

**Figure 12:**
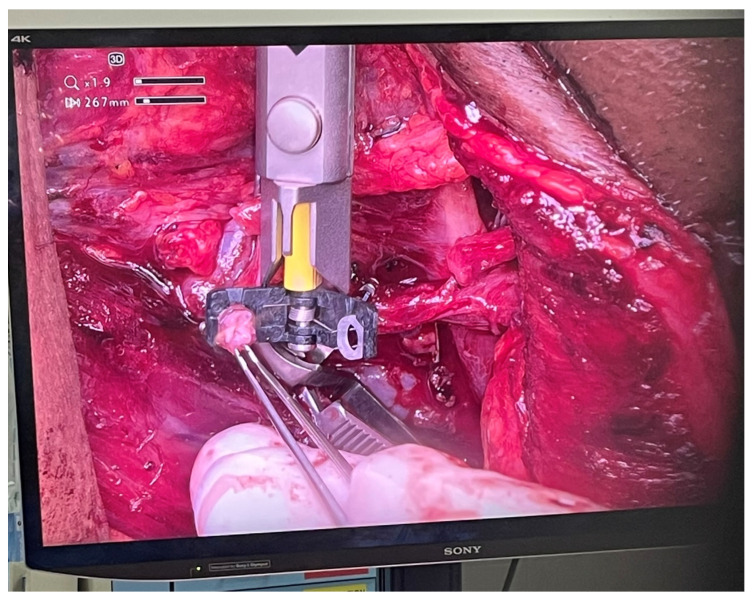
Case 4—Orbeye 4K 3D Camera visual display.

**Figure 13:**
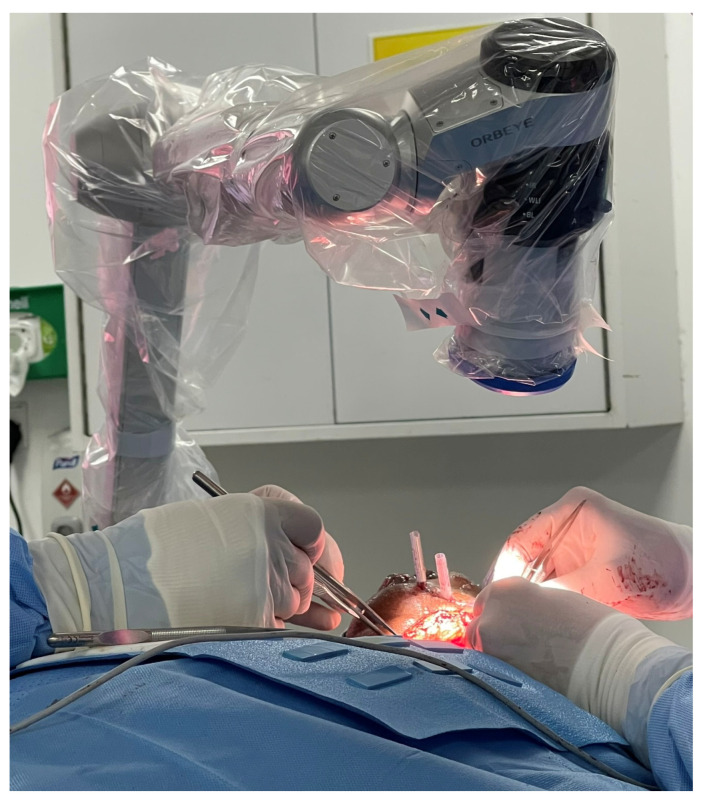
Case 4—Intraoperative Orbeye overhead camera position.

**Figure 14:**
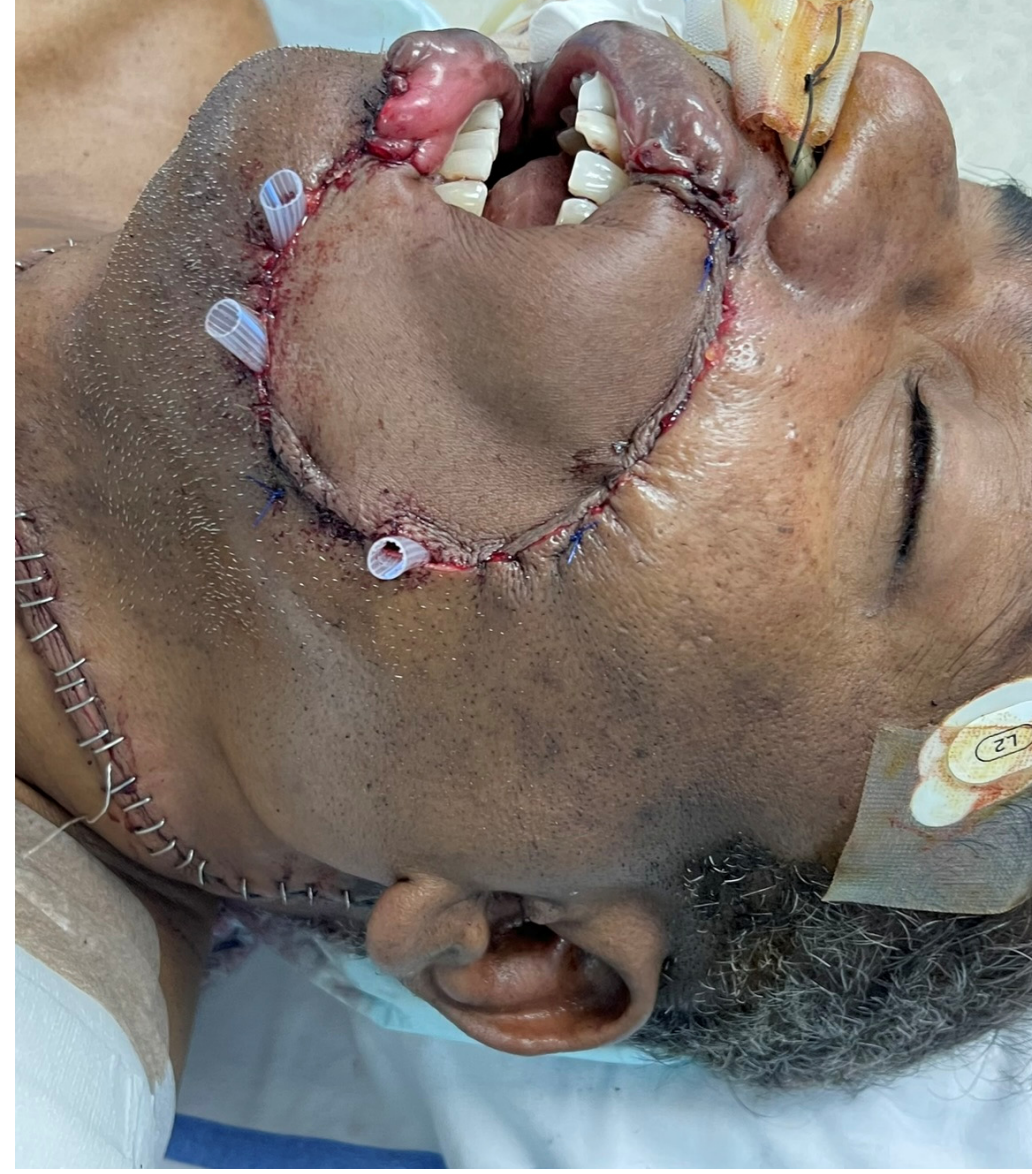
Case 4—Immediate postoperative status.

A 58-year-old male with pT2N0M0 buccal/commissural squamous cell carcinoma underwent composite resection and radial forearm free flap reconstruction in August 2024. Cervical microvascular anastomoses were completed using the Olympus Orbeye 4K 3D exoscope camera system (Olympus Deutschland GmbH, Hamburg, Germany). Implantable Cook microdoppler dual-channel monitoring system was utilized perioperatively to ensure microvascular patency and flow (Figure 11, Figure 12, Figure 13 and Figure 14).

### 3.5. Case 5

**Figure 15:**
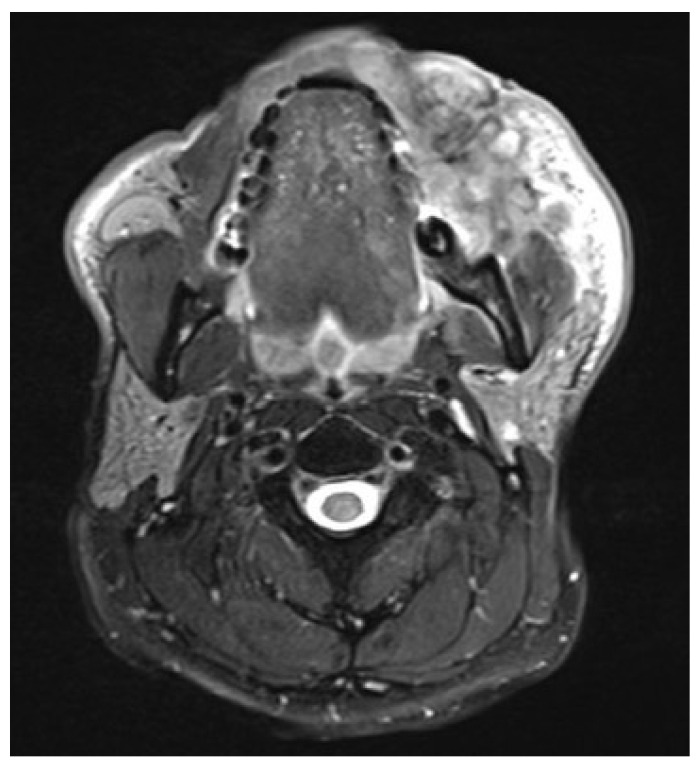
Case 5—Preoperative staging T2W magnetic resonance imaging (axial view).

**Figure 16:**
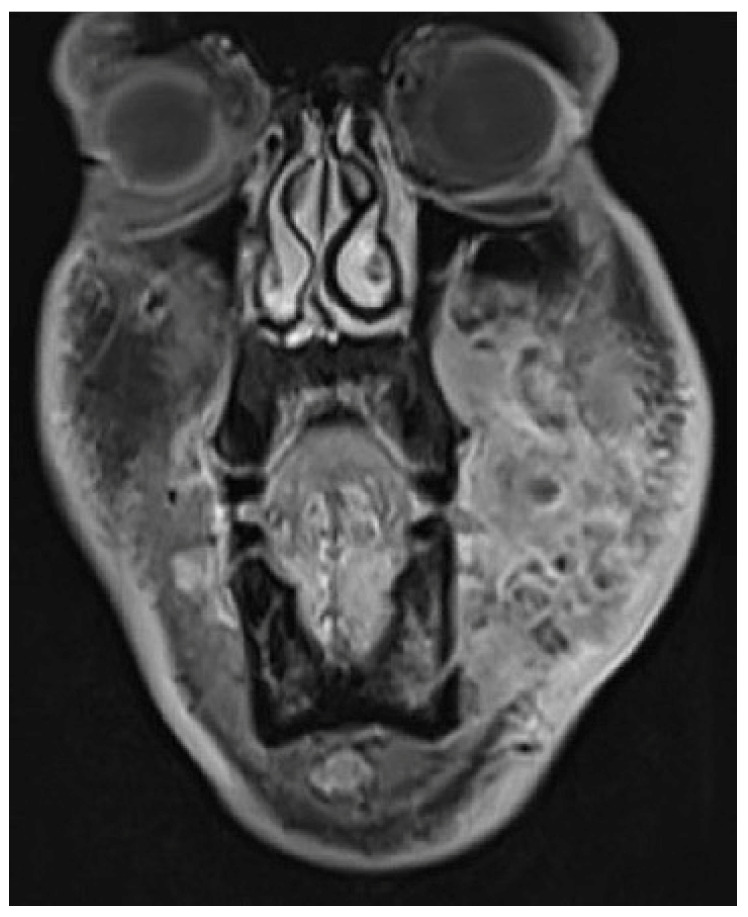
Case 5—Preoperative staging T2W magnetic resonance imaging (coronal view).

**Figure 17:**
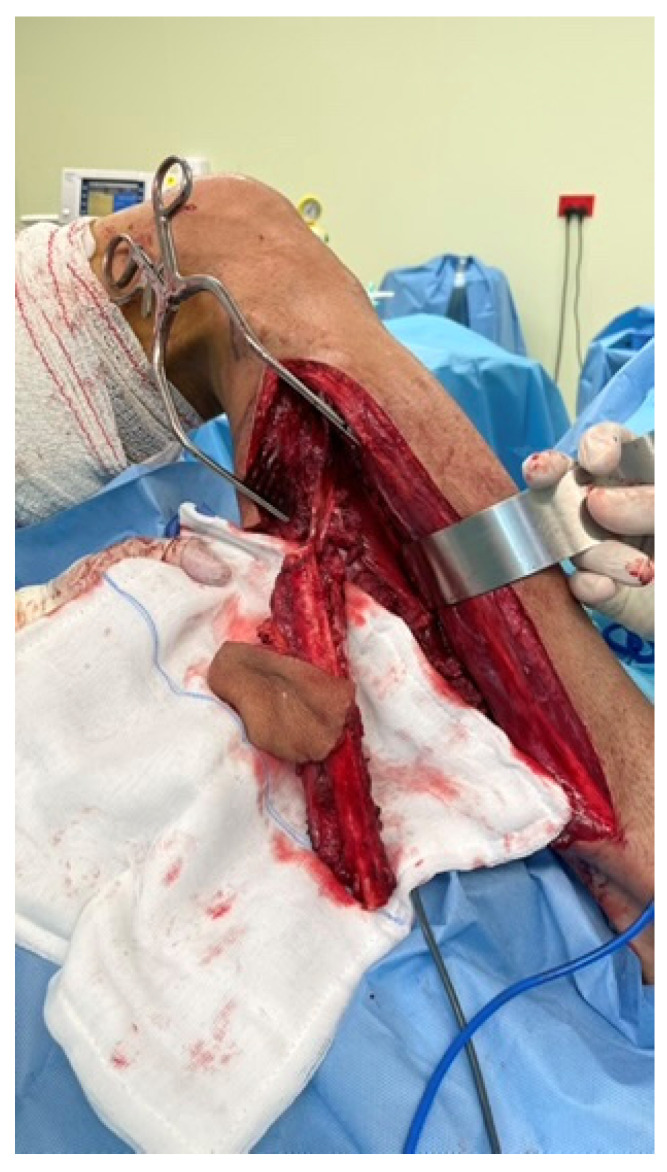
Case 5—Completed fibula flap harvest.

**Figure 18:**
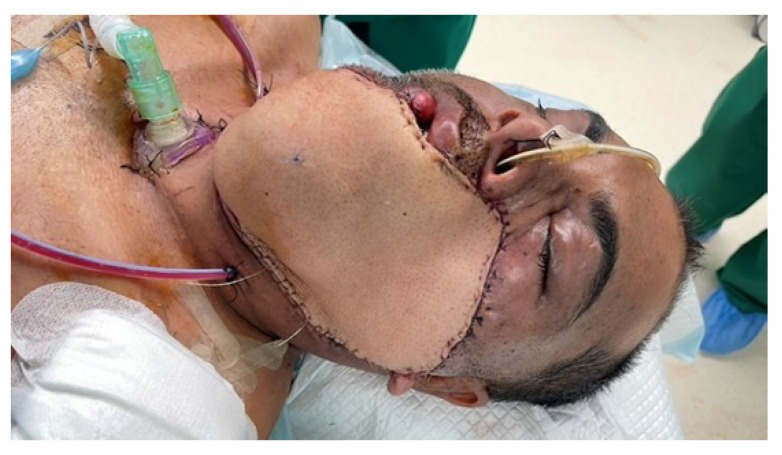
Case 5—Completed cervicofacial reconstruction.

**Figure 19:**
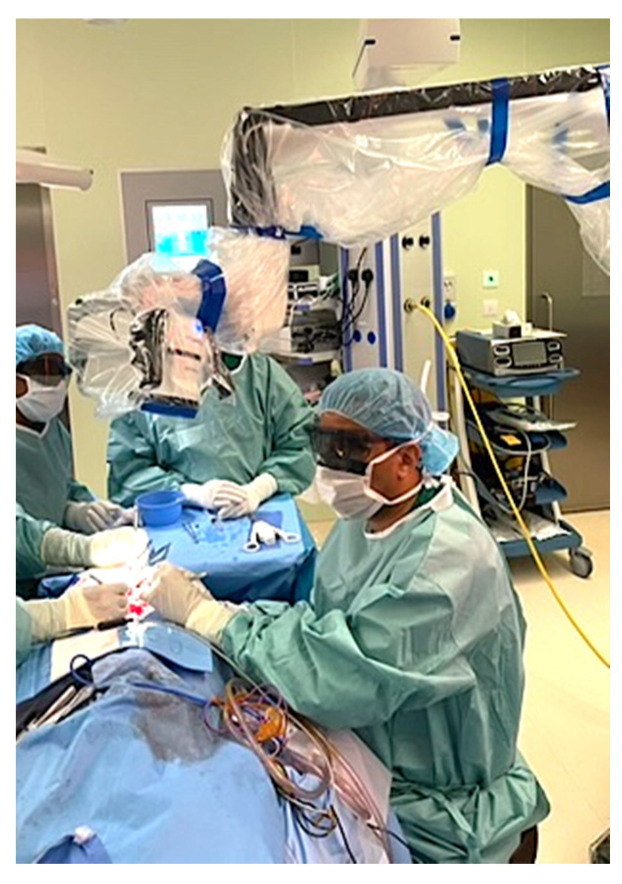
Case 5—Intraoperative exoscope setup during microsurgery.

A 36-year-old male with locoregionally advanced pT4aN2cM0 PDL-1 positive bucco-cervical/mandibular well-differentiated keratinizing squamous cell carcinoma underwent composite resection involving mandible, cheek and cervical skin in December 2024. Dual free flap reconstruction was achieved using free fibula osseomyocutaneous as well as anterolateral thigh perforator flaps. Microsurgery was completed using both Mitaka Hawksight exoscope as well as Zeiss Pentero (Carl Zeiss Meditech AG, Oberkochen, Germany) operating microscope (Figure 15, Figure 16, Figure 17, Figure 18 and Figure 19).

## 4. Discussion

The term exoscope is derived from the Greek exō (“outside”) and skopeîn (“to look”), reflecting its role as an external visualisation device. It functions as a high-definition optical system that provides magnified real-time views of the surgical field on a 3D and/or 4K monitor. Currently available systems, including the VITOM 3D (Karl Storz SE & Co. KH, Tuttlingen, Germany), ORBEYE 4K 3D (Olympus, Germany), and Modus V (Synaptive, Canada), offer high-quality illumination, focus control, and increased flexibility in surgeon positioning [5,11,12].

Although originally developed for neurosurgical procedures [4], exoscopy has gained wider application in craniomaxillofacial and reconstructive surgery. Previous reports have demonstrated comparable outcomes between exoscope-assisted and operating microscope-assisted microvascular reconstruction, with similar rates of vessel patency and flap survival [7,8,9,10]. An additional advantage frequently reported is the ability for the wider operating team to share the same operative view, which may improve communication and facilitate teaching during complex procedures [13,14].

Ergonomics has become an increasingly relevant consideration in microsurgical practice. Prolonged use of the operating microscope is known to require sustained non-neutral postures, and this has been associated with a high prevalence of work-related musculoskeletal symptoms among microsurgeons, particularly affecting the neck and lower back [1,2]. Exoscopic systems remove the need for fixed oculars and allow the surgeon to work in a more upright position, which may help address some of these issues. In the present series, no formal ergonomic assessment tools were used; however, from the surgeons’ perspective, exoscopy allowed greater flexibility in positioning and was perceived to be more comfortable during prolonged microsurgical tasks.

An additional potential advantage of exoscopic visualisation relates to surgical training and performance, particularly among less experienced surgeons. The shared high-definition operative view allows trainees to observe microsurgical steps in real time and facilitates closer supervision during critical phases of reconstruction. Previous work in microsurgical free flap surgery, including studies involving radial forearm free flap anastomosis, has suggested that exoscopic systems may support performance and help shorten the learning curve for junior surgeons when compared with conventional microscope-based approaches [8]. Assessment of surgeon performance was beyond the scope of the present case series; however, this remains an important area for future study.

There remain recognised limitations to the wider adoption of exoscopic systems, including cost, the need to adapt to digital depth perception, and potential visual fatigue associated with prolonged use of 3D displays [7,9]. Despite these challenges, the combination of shared visualisation, workflow flexibility, and perceived ergonomic benefits makes exoscopy an appealing alternative to the conventional operating microscope for selected reconstructive procedures [12,14]. As with much of the existing literature, the present report is limited by small numbers, and larger multicentre studies will be required to better define long-term outcomes and the role of exoscopy in routine microsurgical practice [7,8,12].

## 5. Conclusions

Exoscopic microsurgery is increasingly being used in craniomaxillofacial and head and neck reconstruction and offers a practical alternative to the conventional operating microscope in selected cases. In our experience, exoscopic systems provide high-quality visualisation while allowing greater flexibility in surgeon positioning and facilitating shared viewing for the wider operating team. Although cost, adaptation to digital depth perception, and prolonged use of 3D displays remain relevant considerations, exoscopy was found to be feasible for microsurgical reconstruction across a range of complex procedures. The present case series is limited by small numbers and its descriptive nature; however, it supports the growing body of literature suggesting a role for exoscopy in modern microsurgical practice. Exoscopic visualisation may also offer advantages in training environments by facilitating supervision and shared operative viewing, particularly for junior surgeons undertaking microsurgical reconstruction.

## Data Availability

The data presented in this study are available on request from the corresponding author. The data are not publicly available due to ethical and privacy restrictions involving patient information.
